# Molecular Functionality of Plant Proteins from Low- to High-Solid Systems with Ligand and Co-Solute

**DOI:** 10.3390/ijms21072550

**Published:** 2020-04-06

**Authors:** Vilia Darma Paramita, Naksit Panyoyai, Stefan Kasapis

**Affiliations:** 1 Department of Chemical Engineering, State Polytechnic of Ujung Pandang, Tamalanrea, Makassar 90245, Indonesia; vilia.paramita@gmail.com; 2 Department of Agroindustry, Rajabhat Chiang Mai University, Chiang Mai 50330, Thailand; naksit_pan@cmru.ac.th; 3 School of Science, RMIT University, Bundoora West Campus, Plenty Road, Melbourne, VIC 3083, Australia

**Keywords:** plant proteins, low- to high-solid systems, diffusion, protein-ligand interactions

## Abstract

In the food industry, proteins are regarded as multifunctional systems whose bioactive hetero-polymeric properties are affected by physicochemical interactions with the surrounding components in formulations. Due to their nutritional value, plant proteins are increasingly considered by the new product developer to provide three-dimensional assemblies of required structure, texture, solubility and interfacial/bulk stability with physical, chemical or enzymatic treatment. This molecular flexibility allows them to form systems for the preservation of fresh food, retention of good nutrition and interaction with a range of microconstituents. While, animal- and milk-based proteins have been widely discussed in the literature, the role of plant proteins in the development of functional foods with enhanced nutritional profile and targeted physiological effects can be further explored. This review aims to look into the molecular functionality of plant proteins in relation to the transport of bioactive ingredients and interaction with other ligands and proteins. In doing so, it will consider preparations from low- to high-solids and the effect of structural transformation via gelation, phase separation and vitrification on protein functionality as a delivery vehicle or heterologous complex. Applications for the design of novel functional foods and nutraceuticals will also be discussed.

## 1. Introduction

For some time now, the use of proteins and peptides as multifunctional ingredients with physiological and nutritional benefits has been actively researched. Increasingly, however, animal-based proteins are of growing concern due to potential meat-transmitted disease [1], meat-intolerance leading, for example, to abdominal pain or cramping [2], and dairy allergies [3]. Plant-based biomaterials are often chosen over animal products as a good source of minerals, vitamins and protein in diet, in addition to ethical, environmental or religion/cultural beliefs/concerns. Although, plant proteins are sometimes perceived as lacking optimal textural properties, they are preferred over the animal-based proteins due to safety or health considerations, biocompatibility and sustainability of production [4].

Many studies have been conducted on plant proteins, in order to overcome structural drawbacks and achieve properties as the animal counterparts in the design of emulsifiers and stabilisers [5]. The results indicate that the bio- and techno-functional properties of plant proteins can be improved through processing, including by heating, crosslinking with natural materials (e.g., genipin) [6] and binding with other biomaterials, like phenolic compounds. Specific treatments have been employed, including ultra-high temperature (UHT), high pressure and ultrasonication, to manipulate the structural behaviour and interactions with bioactive compounds/drugs leading to comparable functionality with corresponding dairy protein and Bovine Serum Albumin (BSA) systems [7,8,9,10].

An effort has been made to relate the changes in physicochemical and mechanical properties to the protein source and its amino acid composition. It was confirmed that the stabilisation of protein networks requires an unfolding and realignment of the secondary structure, assisted by the formation of disulfide bridges, hydrogen bonds and hydrophobic interactions [11]. Distinct variations in the proportion of α-helix and β-sheet was recorded upon thermal and non-thermal treatment, with aqueous solutions at low and intermediate levels of solids (< 50% *w/w*) being more susceptible to processing, as compared to the high solid pastes (80% *w/w*) of soy glycinin [12]. In the case of pea protein isolates, heat treatment induced molecular denaturation that increased the β-sheet and reduced the α-helix and random coil contents. The addition of plasticizers altered water binding capacity and improved the overall mechanical properties of pea protein films [13,14]. Ground nut protein concentrates and oat-based gels were also evaluated in terms of their oil binding capacity, emulsifying/foaming property, mechanical characteristics and sensory perception in an effort to increase the consumer acceptability and plant protein intake in the diet [15,16].

Elucidation of the structure-function relationship of proteins allows their utilisation as excipients in various platforms, namely micro- or nanoparticles, beads, hydrogels, electrospun fibres and films for medical applications [4]. Incorporating drugs in the protein matrix creates delivery vehicles whereby the electrical charge, polarity or hydrophobicity of the side groups of the protein are used, in order to manipulate the interactions with the synthetic bioactives and regulate their transport. Such work has been carried out on zein, pea and rapeseed films and nano/micro-carrier systems [17,18]. Animal origin proteins, including whey protein, casein, β-lactoglobulin and gelatin have been utilised, of course, extensively as delivery matrices of various hydrophilic bioactives, including vitamin B3 [19], vitamin B6 [20], vitamin B9 [21], anthocyanins [22] and curcumin [23], and hydrophobic bioactives such as Vitamin D3 [24], Vitamin E [25], and omega-6 fatty acids [26].

Cuq, Gontard, & Guilbert [27] discussed the use of plant proteins as packaging materials and, to some extent, Wan, Guo and Yang [28] their application in the controlled delivery of bioactive ingredients. Five years on, it is desirable to summarise and report on the latest developments in this rapidly evolving field. This includes the molecular understanding of plant-protein morphology and transport phenomena, as well as plant protein-ligand interactions. In the present review, therefore, we shall attempt to explore the actual knowledge concerning the relationship among gel formation, phase separation and vitrification of plant protein matrices and controlled release of bioactive ingredients in added value food systems. The data is complemented by a discussion of the interaction between plant protein molecules and ligand as a function of thermal and non-thermal processing.

## 2. Structural Functionality of Plant Proteins from Low to High Solid Systems

### 2.1. Effect of Heating

Heating is the main physical factor to induce gelation in globular proteins, thereby, altering their functional properties dramatically. Upon heating, native globular molecules in aqueous solution unfold and expose the hydrophobic core facilitating polypeptide-polypeptide and polypeptide-water interactions [29]. This process is initiated by diminishing stability of hydrogen bonds in the hydration shell of the protein, which enables the rearrangement of chain segments to balance a multitude of attractive and repulsive forces [30]. At high enough concentrations, gels are formed upon cooling via disulfide, ionic, hydrophobic and hydrogen bonds [31].

Plant globular proteins, extracted from legume seeds, exhibit structure formation as for the animal counterparts with some variation according to their physicochemical fingerprints [32]. Thus, the β-sheet content of globulins in soy, kidney bean and field pea protein isolates relates positively to the small deformation properties of firmness and yield strength [12,33]. Large deformation properties depend primarily on the number of disulfide bridges. For example, the 2S fraction of soy protein produced flexible structures with reduced water-holding capacity than the 7S fraction due to less disulfide bridges [34]. The high content of disulfide bridges in native 11S fraction of lupin protein isolates prevented molecular reconfigurations upon moderate heat treatment [35]. At the isoelectric point (pH 4–5), aggregation of globulins extracted from soy, quinoa and sesame occurred since the ionization of amino-acid side chains is reduced. Heating can disrupt the disulfide bridges and in alkaline conditions, protein solubility is encouraged due to increased electrostatic repulsion [36,37,38].

Plant protein blends, with other biopolymers, are of interest in novel product development and in understanding the thermodynamic or kinetic processes, as a function of extrinsic factors, including temperature, pH and ionic strength, facilitates structural and functional manipulation. In a relatively dilute aqueous solution, plant proteins show co-gelation with animal proteins [39] or independent gelation that traces the structural characteristics of each component in the mixture [40]. This was shown in the synergistic enhancement of storage modulus, gel hardness and paste viscosity for a co-gelated 10–22% (*w/v*) pea protein with whey protein system [39] and phase separated 10–24% (*w/v*) soy protein isolate with gelatine system [40]. Gelation of two molecular fractions of soy protein, 11S and 2S in acidic conditions, showed that the high molecular weight fraction formed the continue phase supporting the discontinuous inclusions of the 2S fraction. Increasing concentrations of the latter were able to change dramatically the gelation temperature, network strength of the composite and the distribution of solvent between the two polymeric phases [41]. Heating soy or pea protein in mixture with micellar casein, in the presence of calcium ions, resulted in the formation of cohesive structures due to the “beneficial” distribution of the counterion within the two proteinaceous phases of the mixture [42].

Increasing additions of globular protein in processed food formulations creates condensed systems where the structural performance is primarily governed by the property of water molecules as plasticizers, as opposed to solvent in low-solid preparations [12,29]. In the case of soy glycinin (11S), a total solids content between 10 and 70% (*w/w*) retained the secondary structure of the polymer, which was mainly β-sheet followed by random coil, β-turn and α-helix. As expected, higher protein concentrations entrapped efficiently water molecules leading to the formation of firm gels [12]. At 80% (*w/w*) solids, soy glycinin exhibits mechanical properties of an amorphous network characterised by low molecular mobility with high strength and brittleness. These mechanical properties are reminiscent of the viscoelasticity of synthetic amorphous polymers and can be treated as such.

Within a certain temperature range (mainly at subzero temperatures) the condensed protein system (80% solids) records high values of shear storage modulus expected for a glassy consistency. In this supercooled region, there is limited molecular mobility of the amino acid backbone and side chains. Structural relaxation of the condensed matrix with heating generates free volume within the entangled polymer chains, with the material undergoing a glass transition indicated by the so-called “glass transition temperature, *T_g_*”. On further heating, the mechanical properties of the high-solid glycinin matrix exhibit a rubbery consistency and the material softens progressively. Vitrification phenomena of condensed glycinin systems follow qualitatively the transition from the glassy to the rubbery state of high-solid globular proteins from animal sources. This allows comparisons to be made of the structural functionality for partial/total replacement of meat proteins in high-solid formulations [12,29].

### 2.2. Effect of High Pressure

High pressure is the most popular non-thermal treatment in the food industry for the modification of the structural properties of materials, without compromising their bio-functionality. Electrostatic bonds are very labile, hydrophobic interactions are easily disrupted and oxidation of sulfhydryl groups is favoured through the application of high pressure processing (HPP) on proteins [43]. This is accompanied by an increase in surface hydrophobicity and formation of disulfide bridges known as protein denaturation leading to aggregation and eventual gelation [44,45,46]. In relatively dilute systems of amaranth protein isolate (5%, *w/w*), HPP at 200 MPa induced partially unfolding of the protein structure and generated some free sulfhydryl groups [7]. The same pressure intensity increased the water-holding and oil-binding capacity of peanut protein isolate [47]. Extensive conformational changes were recorded upon high pressure application (400 MPa) on amaranth solutions resulting in protein aggregation and the formation of biofilms [45]. Seed and legume protein pressurisation up to 600 MPa showed an excessive formation of high molecular weight and large particle-size protein aggregates, yielding reduced protein solubility in aqueous solutions.

The utilisation of high pressure processing as a method of textural variation requires an understanding of the physicochemical environment, with the ionic strength of solutions featuring prominently. The addition of monovalent ions (Na^+^), and especially divalent ions (Ca^2+^ and Mg^2+^), increases the electrostatic interactions, thereby, inducing a desirable aggregation in the preparation of sweet potato protein [48]. This is due to the binding of positively charged salt ions and negatively charged amino acids of the protein molecule, which is enhanced by HPP at 400 MPa, leading to an improvement in the textural properties of salt-added potato protein in formulations. Care should be taken, though, given that over-salting food could adversely affect the textural behavior following pressurisation, for example, by reducing the hardness, springiness and chewiness of plant protein gels, due to the loss of hydrogen bonding between water molecules and over-aggregated protein [49]. In the case of soy protein isolate, added divalent ions at a neutral pH and pressure of 600 MPa exhibited an increase in solubility ranging from the least effect on ferrous ion-beta conglycinin complex to the highest solubility upon calcium chloride addition [49]. In alkaline solutions, soy protein aggregates in the presence of magnesium or calcium ions could be solubilized after high pressure treatment.

A molecular study of conformational transformation at low levels of soy protein (1%, *w/w*) and acidic environment (pH 3), following pressurisation at 600 MPa, was carried out by Puppo et al. [50]. The macromolecular characteristics of the pressurized soy protein recorded an increase in surface hydrophobicity, and a decrease in free sulfhydryl content and thermal stability. These outcomes suggested molecular unfolding, with the secondary structure of glycinin turning from a major α-helix in the native state to mainly β-sheets and random coils following application of high pressure. The dissociation of 11S glycinin and 7S β-conglycinin fractions, treated with high pressure, almost disappeared in enthalpy scans, revealing total protein denaturation that supports the aforementioned thermal stability. These structural changes in the glycinin molecule under high pressure affected its gelation and emulsification patterns [51,52].

**Figure 1:**
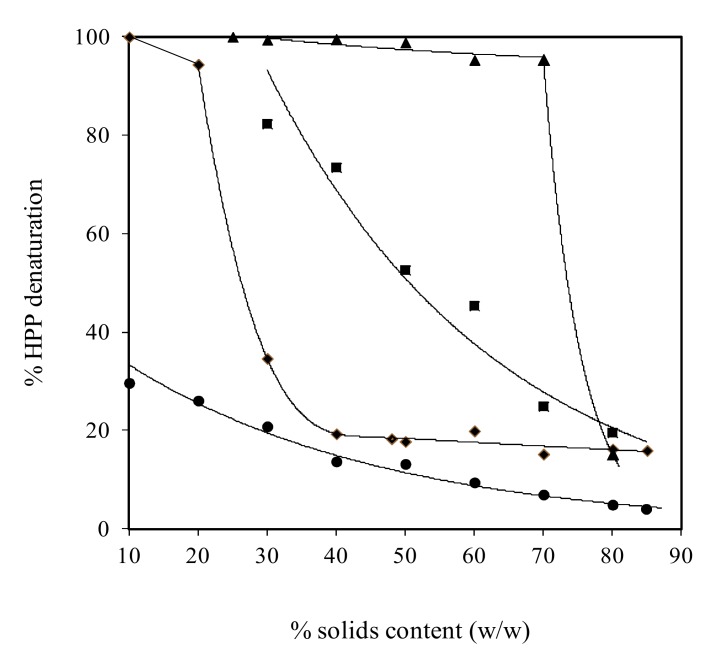
Effect of solids content on the denaturation of pressurised (600 MPa for 15 min) whey protein (▲), soy glycinin (■), ovalbumin (♦) and BSA (●) calculated from denaturation enthalpy (with permission from Savadkhoohi & Kasapis [10]).

Soy glycinin was also examined as part of a wider study on the molecular functionality of globular molecules, including whey protein, ovalbumin and BSA, following pressurisation at 600 MPa for 15 min. As shown in Figure 1, this source of plant protein remained largely in the native state (about 20% denaturation) in condensed preparations of 70–80% (*w/w*) solids that parallels the behaviour of the atmospheric (nonthermally treated) counterparts. The denaturation behaviour of soy glycinin is in between that of whey proteins undergoing almost complete denaturation up to 70% (*w/w*) solids and BSA that retains native conformation due to the presence of seventeen disulphide bridges in the molecule [10]. Despite the preservation of native conformation, soy glycinin at 80% (*w/w*) solids is able to form coherent networks exhibiting comparable mechanical strength and glass transition temperature to that of the thermally denatured counterparts, arguing that pressurisation provides both structural functionality and bioactivity in formulation engineering [12].

### 2.3. Effect of Ultra-High Temperature (UHT) Processing

**Figure 2:**
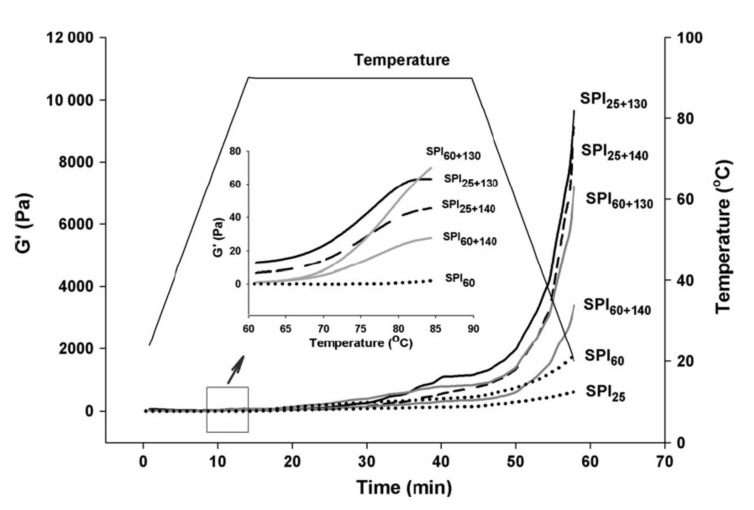
Typical curves of elastic modulus (G′) of soy protein isolate subjected to isothermal and two-stage heating processes plotted against temperature (heating, holding and cooling). The subscript values of 25, 60, 130 and 140 denote heating temperatures (°C) with a holding time of 1 h at 25 and 60 °C (LT), or 4 s at 130 and 140 °C (UHT). The inset magnifies the onset of gelation with rising temperature (with permission from Jian et al. [8]).

This is a hydrothermal process at the elevated temperature of 135–145 °C and a short time of 2–10 s [53] that can improve the solubility [54] and then the gelling capacity [55] of proteins alongside food sterilisation. Jian et al. [8] reported improvement of the solubility from 10 to 95% of soy protein isolate in a two-stage processing, i.e., a low thermal treatment (LT) at 60 °C for 1 h, followed by a UHT treatment at 140 °C for 4 s. The LT treatment enabled the formation of aggregates, held together by non-covalent interactions, while UHT induced primarily the formation of disulphide bridges. This temperature increase, combined with shearing due to steam injection, disrupts various insoluble structures, but encourages the aggregation of 7S and 11S molecular fractions [54]. Figure 2 illustrates dramatic changes in the elastic properties of soy protein in a small-deformation oscillatory test used to confirm the process of gelation, following the aforementioned double-thermal treatment (LT + UHT). Heating and equilibration at 90 °C for 30 min followed by cooling to 20 °C resulted in a significant increase in elastic modulus (G′) that exceeded fivefold the mechanical strength of the soy gel subjected only to LT treatment. It should be noted, however, that in pea protein isolate (PPI), a relatively low solubility (up to 55% of an 8% *w/w* preparation at pH 7.5) has been reported following a similar UHT treatment caused by the presence of insoluble non-covalent aggregates [56].

Plant proteins from cereals, legumes and nuts have been subjected to high pressure homogenization prior to UHT treatment in an effort to reduce their particle size known as ultra high pressure homogenization (UHPH) at 300 MPa [57,58,59]. UHT treatment (140 °C, 2 s) following low-pressure homogenization at 50 MPa (UHT + LPH), autoclave sterilization (AC) at 121 °C for 15 min, and ultra high-pressure homogenization with low thermal treatment at 300 MPa and 50 °C (UHPH + LT) unveiled the physical profile of soy milk. This focused on particle size, degree of protein denaturation, aggregation rate and gel-network density index, which could improve the sensory profile of preparations [60]. Similar outcomes were reported for pea protein isolate, including a UHPH + UHT treatment (500 bar, 140 °C, 2 s) by Qamar, Bhandari and Prakash [61].

In addition, UHT affects the Kunitz trypsin inhibitor in soybeans, a potent protease inhibitor to trypsin and chymotrypsin binding in the human gut. High temperature application (135–150 °C) for 10–50 s inactivated the trypsin inhibitor due to protein aggregation, which is further increased in the presence of sodium chloride [62,63,64]. It was reported that 90% of trypsin inhibitor could be destroyed by a single step of UHT at 143 °C, 60 s or a two-step process of UHT at 143°C, 4 s following low-temperature long-time heating (95 °C, 60 min), with the treated soymilk possessing highly acceptable colour, flavor and vitamin content [65]. The mechanism of UHT-induced inactivation of Kunitz trypsin inhibitor is attributed to alterations in the conformation of the protein by increasing the content of disulfide bonds and non-covalent interactions. In comparison, Bowman-Birk inhibitor, another trypsin inhibitor in soybean, has a higher heat stability to UHT, based on a molecular structure of seven disulfide bonds in the highly hydrophilic protein. The tight conformation diminishes the thermal aggregation of Bowman-Birk inhibitor, leading to loss of bioactivity, but sodium chloride addition can disturb various molecular associations causing inactivation of the inhibitor [62].

With respect to the development of an unpleasant sensory profile in plant protein systems, lipoxygenase is an enzyme that interacts with off-flavour precursors, mainly unsaturated fatty acids, resulting in lipid oxidation. Numerous volatile compounds, e.g., alcohols, aldehydes, ketones and esters are generated, and are capable of deteriorating the organoleptic property of soy protein at low concentrations (1–5 ppb) [66]. Soybean protein is naturally bound to lipoxygenase via non-covalent interactions, and how-temperature heating activates the conformation of the enzyme to act on linoleic acid and initiate off flavour development [67,68]. UHT processing can diminish off-flavour oxidation processes due to enzyme inactivation and soy protein denaturation. That was reported in plant-based dairy substitutes utilised in the manufacture of liquid breakfast that was treated at 145 °C for 8 s and sampled over a short shelf-life of 90 days at 20 and 30 °C [69].

### 2.4. Effect of Ultrasound Treatment

Ultrasonication is a preservation tool that aims to minimise processing but at the same time increase quality and safety of food products [70]. Low-energy (low power, low intensity) ultrasound has frequencies higher than 100 kHz at intensities below 1 W/cm^2^ while high-energy (high power, high-intensity) ultrasound uses intensities higher than 1 W/cm^2^ at frequencies between 20 and 500 kHz [71]. In plant protein studies, low frequency ultrasound of 20–100 kHz and power in the range 100–1000 W/cm^2^ has altered the structural property of plant proteins due to strong shear and mechanical forces, occurring in the cavitation phenomenon [72,73]. This phenomenon induces changes in protein conformation by exposing sulfhydryl groups and increasing the hydrophobic patches of the protein surface hence influencing plant protein functionality.

The impact of ultrasound treatment on plant protein isolates depends on two parameters, i.e., ultrasound intensity [9,72] and ultrasound exposure time [73,74,75,76]. High intensity of the ultrasound wave could induce aggregation of numerous plant protein extracts (pea, canola, album seed) suspended in an aqueous solution. Longer sonication period encouraged protein-water interactions resulting in a broad size distribution of fractured microparticles in the protein suspension [9,74,76].

**Figure 3:**
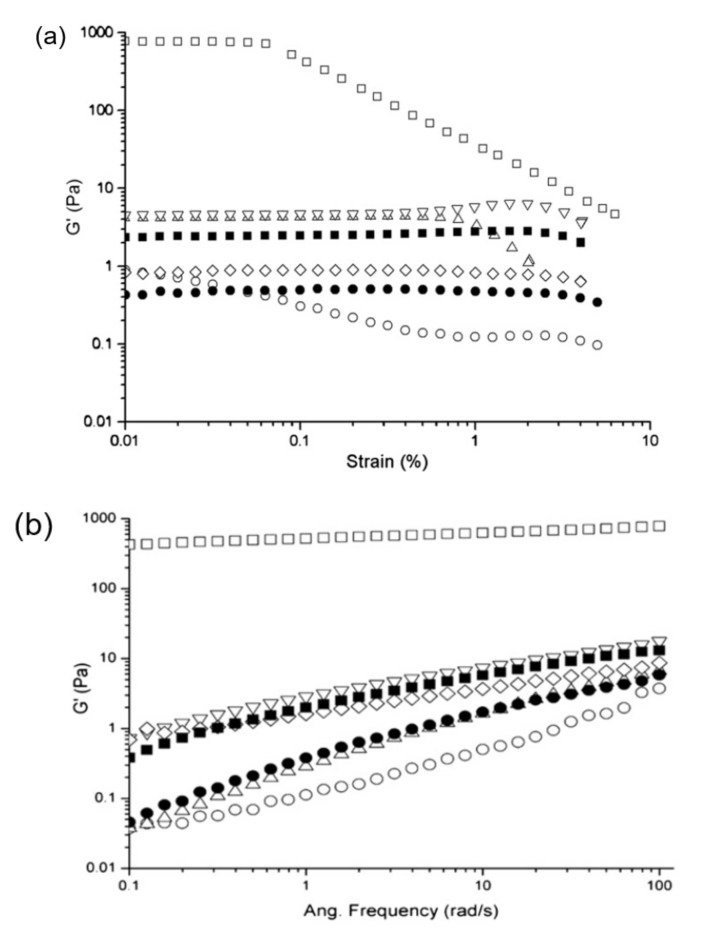

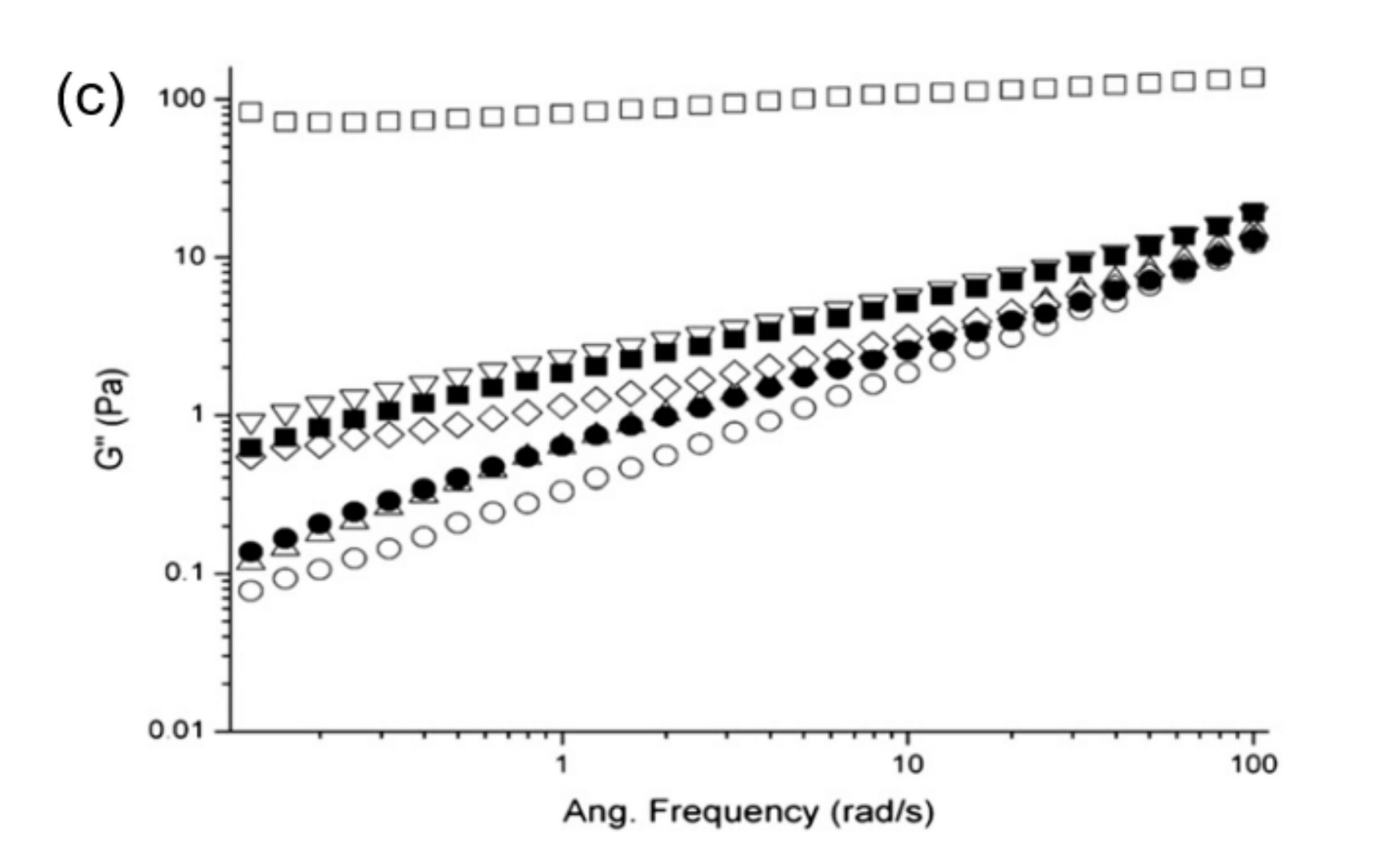
(**a**) Strain sweep, (**b**) elastic modulus (G′), and (**c**) loss modulus (G″) of a 12.5% soy protein dispersion having different ultrasonic treatment of no ultrasound (□); 200W for 15 min (◯); 200W for 30 min (△); 400 W for 15 min (▽); 400 W for 30 min (◇); 600 W for 15 min (■); and 600 W for 30 min (●) (with permission from Hu et al. [77]).

Studies on a soy protein isolate (SPI) dispersion, treated with low-frequency (20 kHz) ultrasonication, at different powers (200, 400 or 600 W) and duration of treatment (15 or 30 min), revealed that longer treatment time and intensity of power reduced the value of elastic modulus (G′) significantly but extended the linear viscoelastic region (LVR) of the rediluted 12.5% SPI dispersion in a strain test (Figure 3a) [77]. Similarly, the value of elastic (G′) and loss (G″) modulus of the ultrasound treated SPI dispersion was lower compared to the non-treated one, accompanied by an order of magnitude increase in moduli from 0.1 to 1 Pa with increasing oscillatory frequency from 0.1 to 100 rad/s. Findings indicated the flow behaviour of SPI with ultrasonic treatment compared to the non-treated sample, with liquid–like properties being more pronounced for the samples treated with low-power (200 W) at a short period of time (15 min) (Figure 3b,c).

On the other hand, ultrasound treatment did not affect the primary structure of numerous legume proteins shown in segmented molecular weight fingerprints analysed using protein gel electrophoresis [9,74,77,78,79]. It can reduce the molecular weight, for example in pea protein (32–55 kDa in Mir et al. [76]) and millet proteins (40–50 kDa in Nazari et al. [72]). However, ultrasonication affected the secondary structure of millet protein isolate, investigated by Fourier-transform infrared spectra (FTIR), zeta-potential values and thermal analysis. The main change in Amide I region (1700–1600 cm^−1^) was reflected in the intensity of C = O stretching vibrations of the peptide bonds in millet protein dispersions of 10% solids. Shift of Amide II bands (1530–1550 cm^−1^) following ultrasound treatment was attributed to the partial transformation of random coil to predominantly β-sheet.

The positional change in Amide II region of millet protein decreased the intermolecular hydrogen bonds, while increased the quantity of negative charges, as recorded by zeta potential values [72]. Disruption of the secondary structure affected thermal stability seen in a decrease in enthalpy of denaturation by DSC heating to 130 °C at 10 °C /min or loss in the weight of protein mass during thermogravimetric analysis by heating to 800 °C at 10 °C/min. In terms of the pea protein dispersion (also 10% solids), electrostatic repulsion showed a converse result to that of millet protein, where the net negative charge was lower in ultrasound treatment due to its distinct secondary structure [9]. In general, the work confirmed the cavitational and turbulent effects of ultrasonication on breaking down high molecular weight into small size particles and the destruction of intermolecular hydrogen bonds in plant protein structure [72,76].

High-solid systems with improved properties were produced using high intensity ultrasound treatment on relatively dilute protein solutions prior to film formation. Ultrasound application allowed the formation of homogeneous protein dispersions via increasing hydrogen bond formation with water molecules. A casting technique was then utilised to prepare thin films at high levels of solids (about 90% *w/w*). In cases of both, gluten and soy-protein based films, the large deformation properties of tensile strength and film elongation were considerably enhanced [80,81]. In addition, composites with a synthetic polymer, polyacrylamide, were prepared in an effort to further manipulate the mechanical properties of soybean protein isolate by varying three experimental conditions: Sonication power, sonication time and slurry temperature [82]. Sonication treatment power varied from 200 to 600 W and generated variable cavitation and microstreaming to rearrange and unfold the soy protein molecule. The film structure, produced by the sonication, showed a higher adhesion force and tensile strength but lower film elongation due to increasing stress effects on the soy protein/polyacrylamide interface in the phase separated mixture. It was also observed that application of sonication power at more than 500 W reduced intermolecular cohesion amongst the polymeric constituents in the matrix by disrupting hydrogen bonding, hydrophobic and electrostatic interactions that led to increased polymer/water interactions.

Low-frequency ultrasound has been a potential non-thermal technique for the improvement of the functional properties of plant-sourced proteins achieving quality at parity to that of animal sources [79]. Functionality mainly focuses on colour, solubility, foaming property, emulsification and gelation. Application of an ultrasound wave increased the whiteness of protein isolate from *Chenopodium album* seed due to the destruction of protein-pigment complexation [76]. The solubility of treated black bean protein isolates was also improved by converting large surface area-to-small particle sizes in protein dispersions and exposing polar surfaces [78]. Sonication increased the water holding capacity of protein in Ganxet beans [83], but partially denatured canola protein by exposing hydrophobic regions to support gelation [74].

Several studies showed high foaming capacity and foam stability of flexible plant-protein secondary structures in aqueous solutions due to an intense homogenization via ultrasound treatment, which increased the surface hydrophobicity and protein diffusion in air-water interfaces. Plant protein foam exhibited the characteristics of a thick and cohesive layer covering air bubbles following stabilization with high-intensity ultrasound waves [9,74,76]. Emulsification of soy protein isolates was also enhanced with various ultrasonication protocols. Emulsion droplets were small in comparison to untreated counterparts, allowing the adsorption of protein at oil-water interfaces with a lower interfacial surface tension. In addition, ultrasound-treated soy protein emulsions were subjected to shelf-life studies to show good stability under storage for 28 days [84].

## 3. Protein-Ligand Interactions

### 3.1. Overview of the Kinetic and Thermodynamic Approach on Protein-Ligand Interactions

Ligand-binding macromolecules play an important role in the controlled delivery of drugs and bioactive food compounds. The underlining process of protein binding with ligands includes factors like the specificity and affinity of the interaction [85]. Specificity reflects the capacity of the protein to differentiate highly specific binding partners from less specific counterparts and maintain its affinity to the former, despite their low concentration, compared to the more concentrated less specific ones [85,86]. The kinetics of protein-ligand binding can be described as a reversible time-dependent mechanism [87],

(1) $$P+L\overset{k_{\mathrm{on}}}{\underset{k_{\mathrm{off}}}{⇌}}\mathrm{PL}$$

where, P and L are the protein and ligand molecules, *k*_on_ and *k*_off_ are the kinetic rate constants of binding and dissociation, and the expression can also be written as:

(2) $$\frac{k_{\mathrm{on}}}{k_{\mathrm{off}}}=\frac{\left[\mathrm{PL}\right]}{\left[P\right]\left[L\right]}$$

Molecular interactions might be the outcome of excited-state reactions, molecular rearrangements, energy transfer, ground-state complexation and collisional quenching [88]. Often, they result in luminance (fluorescence) quenching, which is either static or dynamic [89]. Fluorescence emission spectra are commonly utilised to determine the type of quenching with the Stern-Volmer equation [89,90],

(3) $$\frac{F_{o}}{F}=1+k_{sv}\left[Q\right]=1+k_{q}\tau_{o}\left[Q\right]$$

where, $\left[Q\right]\;$ is the ligand concentration, *τ_o_* is the unquenched lifetime of the protein, *k_q_* is the protein quenching rate constant, *k_sv_* is the Stern-Volmer quenching constant, and *F_o_* and *F* are the emission intensities of the protein-ligand system before and after the addition of the ligand [90]. By plotting the emission intensity, *F_o_/F*, against ligand concentration, we expect to obtain a linear relationship with a slope that is equal to the value of *k_sv_* or *k_q_ τ_o_* [88]_._ The *τ_o_* is normally a predetermined value of the unquenched lifetime of the fluorophore, for example, 3.30 ns for β-casein [91], 4.2 ns for riboflavine [92], 6.2 ns for bovine serum albumin, 2.2 ns for IgG, 1.5 ns for lysozyme and 5.5 ns for pepsin [93]. Notably, the Stern-Vomer equation is only suitable for complexes without residual fluorescence, and when this is not a likely outcome, more sophisticated approaches should be adopted.

**Figure 4:**
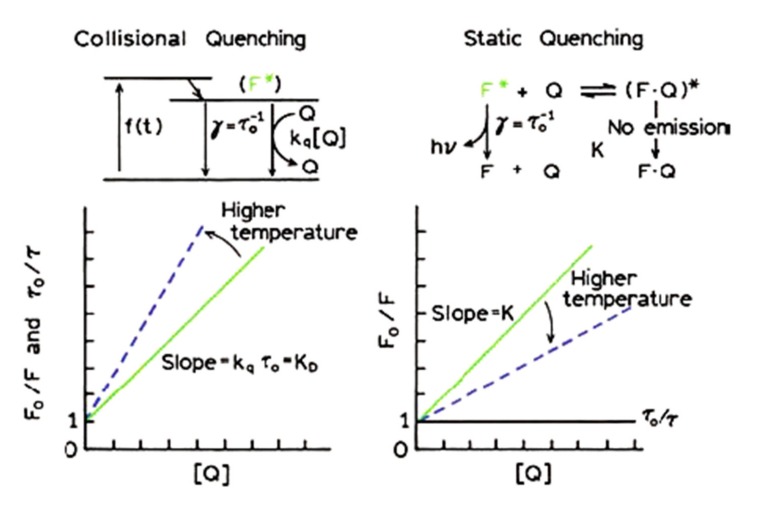
Comparison between static and dynamic (collisional) quenching (with permission from Lakowicz, [88]).

Increases in temperature result in rapid molecular motion and diffusion promoting large collisional events described by dynamic quenching. In contrast, when the temperature increase is followed by the dissociation of the complex (weakening of its bonds) or reduction in *k_q_* values, then quenching is regarded as static (Figure 4; [88]). The association constant (*k_a_*) of the protein-ligand complex and the number of binding sites per protein (*n*) is often determined in literature using the following double logarithmic equation. Again, it should be remembered that (*n*) is the Hill’s coefficient reflecting an index of cooperativity, only yields the binding stoichiometry in infinite cooperativity between protein and ligand, and frequently returns lower values than the real number of binding sites [94,95]:

(4) $$log\frac{F_{o}-F}{F}=logk_{a}+nlog\left[Q\right]$$

It is necessary to document the thermodynamic aspects, in order to further understand the driving force behind the protein-ligand interactions. In spontaneous (exothermic) processes, the binding of ligand to protein is indicated by the negative value of the Gibbs free energy of binding (△G) which is the outcome of variation in the thermodynamic signatures of enthalpy (△H) or entropy (△S)-driven processes [96]. Exothermic reactions relate to the energy of non-covalent interactions, including van der Waals forces, hydrogen bonding, polar and non-polar attractions and ion pairing, between protein and ligand. Endothermic reactions indicate the opposite, i.e. the disruption of molecular bonding [97].

Entropy represents the energetic consequences of changes in the degree of order within a molecular arrangement. Its positive or negative values correspond to the increase or decrease in the degree of freedom of molecular motions [98]. The relationship between △G, △H and △S is given by the classic equation of the Gibbs free energy,

(5) ΔG=ΔH−TΔS

which, can be recast in the following form,

(6) $$\Delta G=-RTlnk_{a}$$

where, *R* is the gas constant (8.314 J mol^−1^ K^−1^), *T* is the temperature (in Kelvin), with the association constant (*k_a_*) being determined from equation (4) [96,98]. To connect the above group of equations (5) and (6), the van’t Hoff equation is used [95]:

(7) $$lnk_{a}=-\frac{\Delta H}{RT}+\frac{\Delta S}{R}$$

From equation (7), values of △H and △S can be easily calculated as the slope and intercept of the linear regression between 1/*T* and ln *k_a_*. Based on this approach, literature suggests that the main binding forces connecting ligand and protein can be identified with the following [99]:

(1) ΔH > 0 and ΔS > 0, mainly hydrophobic/entropic forces
(2) ΔH < 0 and ΔS < 0, van der Waals interactions and hydrogen bonds
(3) ΔH < 0 and ΔS > 0, mainly electrostatic interactions

### 3.2. Pinpointing the Biding Sies with Molecular Docking Simulations

Literature utilises several protocols to identify the nature of interactions between protein and ligand, including spectroscopic, microscopic, thermodynamic, electrophoretic, chromatographic and bioinformatic analyses [100]. We reviewed earlier fluorescence quenching and thermodynamic aspects as suitable probes in this respect. More recently, molecular docking simulations (MDS) show promise in direct elucidation of the specific binding sides being involved in such interactions and the affinity between the two active constituents [101]. In general, molecular docking will examine a range of hypothetical protein structures to decide on the most stable configuration on energetic grounds taking into consideration the non-covalent interactions that stabilise the complex [102].

Molecular docking simulation of various protein-phenolic compounds in Budryn et al. [103] revealed the effect of hydroxyl group methylation and esterification in quinic acid that led to changes in the nature of binding from hydrophobic to hydrophilic. Soy proteins, having a large average peptide length, are able to bind phenolics preferably through hydrogen bonds. Molecular docking simulations follow the so-called “lock-and-key”, “induced fit”, or “conformational selection” [97], with the latter being the most common in plant protein-ligand interactions. In this model, the protein tends to form a flexible structure and the interacting ligand will induce conformational changes in the protein structure towards a state of thermodynamic equilibrium to accommodate the ligand [97]. Currently, there are over 75 types of software available to simulate molecular docking of protein. The types of software include, AutoDock, DOCK, GOLD, ICM, Glide, Surflex, Affinity, LigandFit, Discovery Studio and many others are well-reviewed in the literature [102,104,105]. The most recent docking programs in the last 5 years are CABS-dock, FlexAID, GalaxyPepDock, LightDock and MOLS 2.0 [106,107,108,109,110]. Some popular molecular docking simulations use the CDOCKER protocol that identifies a number of possible binding configurations, known as poses. These are accompanied by energy scores where the lowest value indicates the most stable molecular conformation. For instance, in rice glutelin-resveratrol complexes, the interaction energy ranges from −29.68 to −36.60 kcal/mol for 10 molecular arrangements, and the pose selected was the one with the least energy score within this statistical family (−36.60 kcal/mol) [111].

The favoured configuration provided insights into two possible hydrogen bounding between resveratrol and Gly residues of the rice protein and four hydrophobic interactions between the benzene rings of resveratrol and Phe, Val, Met residues of the rice protein. The results indicated the domination of hydrophobic interactions over the hydrogen bonds in this system [111]. When the same protein was modelled against epigallocatechin gallate (EGCG), it was suggested that the hydroxyl groups of EGCG bind to five amino acids of the protein, mostly with His, Met, Lys, Ser and Gly residues, while the *pi*-alkyl groups of EGCG bind to only three Val groups of rice glutelin through hydrophobic interactions, hence hydrogen bonding dominated over hydrophobic association (Figure 5 in Xu et al. [90]). In rice glutelin-linoleic acid complexes, the association is mainly *via* van der Waals forces with 13 different amino acids at 15 distinct binding sites (Leu, Asp, Glu, Cys, Arg, Pro, Thr, Gln, Gly, Asn, Cys, Ala, and Phe) and two hydrogen bonds with Val and Arg residues [95]. Further details of plant protein-ligand associations are summarised in Table 1.

It should be mentioned that predictions of molecular docking are often checked with thermodynamic parameters obtained from isothermal titration calorimetry (ITC), including values of free energy, enthalpy, entropy and overall heat involved in molecular interactions [100]. A comparison study between soy protein isolate and whey protein concentrate, for instance, revealed that the former has △G of about -23.5 kJ/mol for caffeic and ferulic acid compared to -20.5 kJ/mol for the latter. Outcomes indicate that the soy protein-bioactive interaction is more extensive than whey protein, as confirmed in the number of binding sites in soy protein that were 16 times more for caffeic acid and 2 times more for ferulic acid than whey protein, according to MDS [122]. Both proteins had △H < 0 and △S > 0 indicating dominant electrostatic attractions in these systems. Similar agreement between ITC results and MDS predictions were observed for soy protein hydrolysates in comparison to whey protein hydrolysates [103].

## 4. Transport Phenomena of Bioactive Compounds in Plant Protein Matrices

Plant proteins have been used as an alternative material to animal proteins and synthetic polymers for safe and effective delivery of microconstituents [85,126]. Cereal, legume and seed proteins have been applied in coating a wide range of bioactive compounds including antioxidants, vitamins, drugs, polyunsaturated fatty acids, essential oils, etc. shown in Table 2. Delivery vehicles can take the form of a sphere (capsule), cylinder (tablet, fibre) and slab (film, sheet) with different sizes ranging from nano up to millimeter scale [28,127]. They are designed to control drug release by site-, temperature-, void free volume- or time- dependent events [5,128]. In the case of non-degradable systems, extensive solvent penetration initiates a plasticising effect favouring the release of entrapped molecules. Therefore, the release of essential oils, lauroyl arginate, etc., from slightly-swellable zein protein films was prolonged following the kinetics of Fick’s second law of diffusion [129,130].

Mobility of small molecules is classified as Fickian (Case I) or non-Fickian (Case II) based on the exponent, *n,* of the power law (*M_t_/M_∞_* = *k t*^n^) introduced by Ritger and Peppas [154]. *M_t_* and *M_∞_* are the mass of penetrant at time *(t*) and time approaching infinity, *k* is a structural constant for a particular system and *n* is an exponent representing the release mechanism of active compounds [155]. Fickian diffusion was indicated by a value of the release exponent of about 0.50 or slightly lesser depending on geometry of the delivery vehicle [150]. That was demonstrated in the release of bovine serum albumin from soy protein/polyacrylic acid composite hydrogels. It was rapid, within four hours, reaching equilibrium in the next eight hours without matrix degradation. This release profile of protein cargo was suitable for drug delivery in medical remedy [146,156].

Controlled diffusion of nicotinic and linoleic acids has been demonstrated in high solid systems (>80% *w/w*) of whey protein in microcapsules [19] and slabs [157] using the concepts of glass transition and free volume theory [128,158]. In plant proteins, a blend of soy protein isolate, maltodextrin and gum Arabic was used to encapsulate paprika oleoresin showing increased stability with higher glass transition temperature, *T_g_* [145]. The release kinetics of curcumin from zein electrospun fibres indicated a profile of less Fickian diffusion, with the value of release exponent (*n*) from the power law equation dropping to 0.32 with increasing *T_g_* values of the system from 168 to 172 °C [136]. Carvacrol diffusion from soy protein coated paper with relative humidity varying from 60 to almost 100% indicated the effect of the glass transition temperature and moisture content on the release profile of the bioactive. Transport of carvacrol was two orders of magnitude higher at 30°C compared to 5 °C, i.e., close to the glass transition temperature of the soy protein matrix at RH of about 100% [159].

In the case of swellable systems, changes in mesh size was a critical parameter in the diffusion of bioactive compounds following infusion of water molecules in the polymeric network [128,160]. Swelling resulted in anomalous diffusion, including Fickian (Case I) and polymer relaxation (Case II) contributions, in the transport of a range of water-soluble vitamins [20]. Network morphology of gelatin and BSA was modified by the modulation of chemical crosslinking, e.g., with genipin in Teimouri et al. [20] and Whitehead, Paramita, Teimouri, Young, and Kasapis [161] or physical and enzymatic crosslinking [162]. In plant proteins, barley glutelin, crosslinked with 2.5% added glutaraldehyde, was able to successfully encapsulate β-carotene and then release it with zero-order kinetics in simulated gastric fluids [151]. The release behaviour of the natural antimicrobial compound, lysozyme, from wheat gliadin films, cross-linked with cinnamaldehyde, showed a decrease in the apparent diffusion coefficient from 4.62 × 10^−15^ cm^2^/s with 1.5% crosslinker to 0.06 × 10^−15^ cm^2^/s with 5% crosslinker. The reduction was due to shrinkage of the matrix by 25% [149].

The nature of the protein-ligand interaction was found to regulate the release profile of bioactives in a range of plant protein systems. For example, fruit aroma compounds were strongly bound to soy protein isolate [163] and tea polyphenols were bound to molecular fractions of 7S and 11S soy protein under high pressure treatment at 400 MPa [113]. An increase in the thermal energy of processing or upon product utilisation weakens the complex interactions to promote the release of bioactive compounds. Effect of pH on regulating the transport of urea from wheat gluten membrane was also studied [164]. A rapid diffusion was observed at pH 4, as compared to pH 7 and 10. At acidic conditions, there was an electrostatic repulsion between protein and ligand, which generated free volume/space in the macromolecular assembly encouraging urea release. This process at pH 4 and ambient temperature followed anomalous transport (both diffusion and relaxation contributions) but at pH 7, it was solely determined by the Fickian release of urea [164].

Gliadin and zein are two prolamins from wheat and corn of great interest in drug delivery due to the limited water solubility and good film forming property. The former was used in the entrapment of α-tocopherol molecules and their subsequent release in nitrogen over 100 h at 25 °C and dark ambience. The vitamin released pattern occurred in two-steps, a burst effect, i.e., a rapid release within 60 min due to the weak vitamin interaction with the protein, followed by a slower and well controlled diffusion process. Similarly, an initial burst release was observed in electrospun gliadin nanofibers incorporating ferulic acid and hyroxypropyl-β-cyclodextrin in the formation of active food packaging coatings. Interaction between hydroxypropyl-β-cyclodextrin and ferulic acid blocked the phenyl ring of the latter to provide required stability, *via* hydrophobic interaction, within the prolamin matrix leading to optimum release of the hydroxycinnamic acid in food applications [148]. Finally, a release study on the thymol/γ-cyclodextrin complex encapsulated in zein nanofibrous web showed an increase in the amount of thymol release at temperatures 37 °C to 75 °C due to weakened hydrogen bonds, increase in the mobility of polymer chains and an increase in the kinetic energy of thymol diffusion that served well in antimicrobial food preservation [165].

## 5. Conclusions

Research on plant protein biomaterials aims to develop alternative sources to animal-based proteins and synthetic polymers for food use and drug administration. Thermal and non-thermal processing reveal promising ways of changing the structural characteristics of plant proteins, leading to an improvement in functional properties, including solubility, stability, texture, appearance and flavour release. As excipients for the delivery of bioactive compounds and drugs, plant proteins are preferred over animal counterparts due to medical-related issues, biocompatibility and sustainability of production. However, the quantification of microconstituent release in relation to the morphological characteristics of the plant protein network is well behind similar studies on gelatin, blood-based proteins and polysaccharides. Similarly, complex formation between plant protein and ligand needs to be further explored at parity with meat, egg and milk proteins by establishing the amino acid sequence and secondary conformation of promising plant protein variants. Thermodynamic approaches in combination with computational modelling can assist in simulating binding patterns and predicting energy requirements for complexation and subsequent release of the bioactive compounds servings as a springboard for in vivo studies.

## Figures and Tables

**Figure 5:**
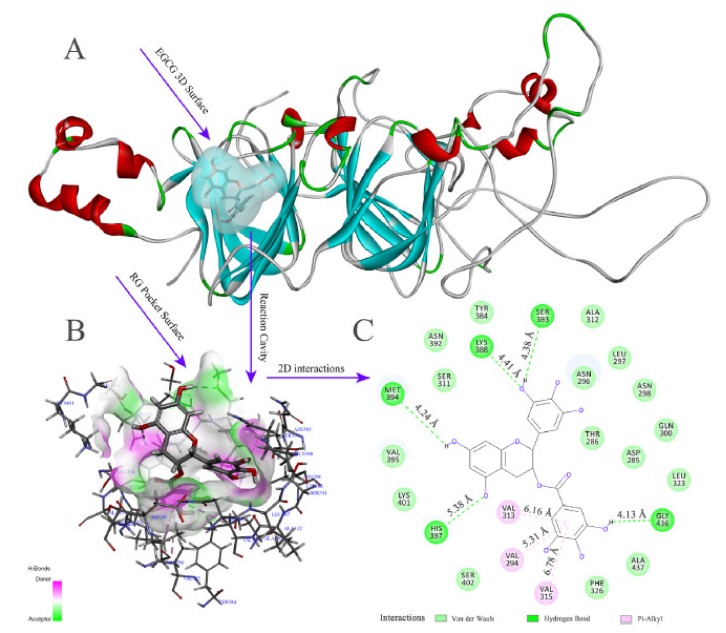
(**A**) Three-dimensional (3D) docking model of rice glutelin (RG)-EGCG interaction simulated by Discovery Studio 3.0 (Accelrys Inc., USA), (**B**) The hydrogen bond surface of protein receptor interacting with EGCG, the blue and green colors represent the donor and acceptor of hydrogen bonds, respectively, and (**C**) Two-dimensional (2D) schematic interaction diagram between EGCG and RG, the color of amino acid residues is drawn by interaction (with permission from Xu et al. [90]).

**Table 1:** Plant protein-ligand interactions.

Protein	Ligand	Method	Main Findings	References
**a. Polyphenols**				
Soy glycinin and soy trypsin inhibitor	Chlorogenic acid, caffeic acid, gallic acid, flavonoids, flavone, apigenin, kaempferol, quercetin and myricetin	FUV-CD IF FLQ DSC	-Secondary and tertiary structure of protein changed (protein surface became more hydrophilic) -Formation of new non-covalent forces (inter- and intramolecular interactions, e.g. ionic/hydrogen bonds) by the introduction of the carboxylic groups (following the covalent attachment of the phenolic acids) and by the hydroxy groups (in flavonoids) with protein, for all samples but flavone, apigenin and kaempferol	[112]
Soybean protein	Tea polyphenol	IF CD FS MDS	-High pressure treatment (400 MPa) increased β-sheet and reduced α-helix and polyphenol protex helix structure -Interactions were mostly hydrophobic and hydrogen bounding with polyphenol binding to the 7s and 11s protein fractions	[113]
Glycinin, β-conglycinin and soy protein isolate	Anthocyanins (mainly CYG and CYR)	FS MDS	-The Trp residue of soy protein shifted to a more hydrophilic environment due to protein-ligand interaction -Glycinin has higher affinity towards CYG and CYR compared to β-conglycinin, but native β-conglycinin can bind one CYG and three CYR molecules simultaneously, while other soy proteins can accommodate one ligand only	[114]
Rice glutelin	Resveratrol	CD FS MDS	Binding of resveratrol to protein was spontaneous and mainly driven by hydrophobic interactions	[111]
Rice glutelin	Gallic acid	CD FS MDS	The hydrogen bonds and van der Waals forces were the main factors affecting protein-ligand interactions which led to conformational changes in the protein structure	[115]
Rice glutelin	EGCG	CD FS MDS	Hydrogen bonding and hydrophobic associations cause changes in secondary structure of protein	[90]
Zein	EGCG, Quercetagetin and chlorogenic acid	FUV-CD FS DSC	Secondary and tertiary structure of protein changed depending on nature of interaction (covalent and non-covalent)	[116]
Zein-BSA-CA conjugate	Resveratrol	FTIR	Protein-resveratrol interactions occur via hydrogen bonds, hydrophobic interactions, or electrostatic interactions	[117]
2S albumins in peanuts	EGCG	CD FLQ ITC	Complex formation followed by change in protein secondary structure	[118]
**b. Flavonoids**				
Soy protein isolate–κ-carrageen	Quercetagetin	CD	-Interaction between the quercetagetin and protein was through the hydrophobic interaction hydrogen bonding	[119]
Zein-CAS NPs	Curcumin	DSC FTIR	Zein-CAS NPs interacts with curcumin via hydrogen bonding and hydrophobic interactions	[120]
Lupin seed globulin	Flavonoid (apigenin glycosides)	SSF FL	-Lupin seed globulins bind with phenolic compounds through electrostatic attraction or hydrogen bonding. Pepsin digestion caused release of apigenin glycosides (mainly 7-O-β-apiofuranosyl- 6,8-di-C-β-glucopyranoside)	[121]
**c. Hydroxycinnamic acid and chlorogenic acids**				
Soy protein isolate	HCA (caffeic, ferulic acids), and CHA (chlorogenic acids) from green coffee	ITC MDS	-Significant proportion of HCA and CHA were bound to proteins through electrostatic, hydrogen bonds and hydrophobic interactions -pH affected binding affinity of ligand to soy protein, reduced as pH beyond pI (4.5) -Complexed ligands with β-cyclodextrin limit their binding to protein	[122]
Soy protein hydrolysates	HCA from green coffee	ITC MDS	-The interactions were mostly hydrogen bonds and electrostatic forces being largely stable under proteolytic digestion -Complex ligands with β-cyclodextrin limit their binding to protein hydrolysates	[103]
**d. Others**				
Soy protein isolate	Black soybean seed coat extract	CD FS	-heat treatment led to protein unfolding and enhanced the binding capacity of protein with polyphenols through hydrophobic interaction	[123]
Soybeen 11s and 7S globulin	Lecithin	FS	-Lecithin changed protein structure and enhanced protein hydrophilicity with the effect being more pronounced for 11S compared to 7S protein -Hydrophobic interaction was the major force affected binding of lecithin to 11S and 7S protein	[124]
Rice glutelin	Conjugated linoleic acid	CD FS MDS	-Binding of conjugated linoleic acid to rice glutelin was spontaneous and mainly driven by hydrogen bonds -Fatty acid interaction led to change in secondary structure of protein	[95]
Rice glutelin	Amylose	CD FS MDS	-Rice glutelin bound with amylose through hydrophobic interactions -Secondary structure of protein changed due to binding of the amylose fraction	[125]

FUV-Far-Ultraviolet, CD-circular dichroism, IF- intrinsic fluorescence spectra, FLQ-Fluorescence quenching, DSC-Differential scanning calorimetry, SSF-Steady-State Fluorescence, FL-Fluorescence Lifetime, FS-fluorescence spectra, FTIR-Fourier transform infrared, ITC-isothermal titration calorimetry, MDS-molecular docking simulations, CAS-caseinate, BSA-bovine serum albumin, CA-caffeic acid, EGCC-epigallocatechin gallate, NPs-nanoperticles, HCA-hydroxycinnamic acid, CHA-chlorogenic acids, SPI-soy protein isolate, CYG-cyanidin 3-glucoside, GYR-cyanidin 3-rutinoside.

**Table 2:** Plant protein matrices for transport of drugs and bioactive compounds.

Protein	Bioactive Compound	Geometry	Bioactive Release Mechanism	Reference
Zein-chitosan complex	α-tocopherol,	NP	Burst release within 1.5 h	[131]
Zein- CMCh	Vitamin D3	NP	Burst release within 1.5 h	[132]
Zein	Docosahexaenoic acid (DHA)	UTC	Not specified	[133]
Zein	LysozymeCatechin	F	Burst release within 1 h	[134]
Zein	Resveratrol	NP	Fickian diffusion within 1 h and erosion/relaxation release process after 3.5 h	[135]
Zein	Essential oil	F	Fickian diffusion	[130]
Zein-BSA-CA conjugates	Resveratrol	NP	Not specified	[117]
Zein-caseinate composite	Curcumin	NP	Burst release within 10 min	[120]
Zein	Curcumin	EF	Fickian diffusion	[136]
Zein/SSPS	Lutein	NP	Not specified	[137]
Zein	Theophylline *	T	Mostly Fickian diffusion with contribution of matrix relaxation based on Peppas-Sahlin equation	[138]
Zein	Glibenclamide *	NP	Fickian diffusion	[139]
Zein	Gentamicin *	MM	Fickian diffusion	[140]
Soy Protein	Ibuprofen *	MP	pH sensitive release	[141]
Soy Protein	Riboflavin	HG	Fickian diffusion	[142]
Conjugated soy protein-Folic Acid	Curcumin	NP	Burst effect within 1 h	[143]
Soy Protein	α-Tocopherol or Ascorbic acid	MC	Not specified	[144]
Soy Protein Isolate	Paprika oleoresin	MC	Not specified	[145]
Soy Protein	Bovine serum albumin	HG	Not specified	[146]
Soy protein isolate and SPI-CMCh	Vitamin D3	NCS	Fast release within 1 h	[147]
Gliadin	Ferulic acid with hydroxypropyl- β-cyclodextrin	EF	Burst release within 10 min	[148]
Wheat Gliadin	Lysozyme	F	Fickian short time diffusion	[149]
Modified rice Proteins with eugenol	Caffeic acid phenethyl ester	NC	First-order release, burst release within 3 h	[150]
Barley Glutelin Crosslinked Glutaraldehyde	β-Carotene	MC	Zero-order release kinetics following enzymatic degradation	[151]
Pea Protein	Conjugated linoleic acid	MC	Not specified	[152]
Oat protein isolate	Riboflavin	B	Non-Fickian transport	[153]

UTC-ultrathin capsules, MC-Microcapsule, MP-Microparticle, HG-Hydrogel, EFElectrospun fiber, F-Film, NP-Nanoparticle, NC-Nanocapsule, MM-Multilayer Membrane, T-Tablet, NCS-Nanocomposite, B-Bead, SSPS-soluble soybean polysaccharide, BSA-bovine serum albumin, CA-caffeic acid, SPI-soy protein isolate, CMCh-carboxymethyl chitosan. * Drugs (other references are food bioactives).
